# The Efficacy of a Haptic-Enhanced Virtual Reality System for Precision Grasp Acquisition in Stroke Rehabilitation

**DOI:** 10.1155/2017/9840273

**Published:** 2017-11-05

**Authors:** Shih-Ching Yeh, Si-Huei Lee, Rai-Chi Chan, Yi Wu, Li-Rong Zheng, Sheryl Flynn

**Affiliations:** ^1^School of Information Science and Technology, Fudan University, Shanghai City, China; ^2^Department of Physical Medicine and Rehabilitation, Taipei Veterans General Hospital, Taiwan and National Yang-Ming University, Taipei, Taiwan; ^3^Department of Rehabilitation Medicine, Huashan Hospital, Fudan University, Shanghai, China; ^4^Blue Marble Game Ltd., Los Angeles, USA

## Abstract

Stroke is a leading cause of long-term disability, and virtual reality- (VR-) based stroke rehabilitation is effective in increasing motivation and the functional performance. Although much of the functional reach and grasp capabilities of the upper extremities were regained, the pinch movement remains impaired following stroke. In this study, we developed a haptic-enhanced VR system to simulate haptic pinch tasks to assist the recovery of upper-extremity fine motor function. We recruited 16 adults with stroke to verify the efficacy of this new VR system. Each patient received 30 min VR training sessions 3 times per week for 8 weeks. Outcome measures, Fugl-Meyer assessment (FMA), Test Evaluant les Membres superieurs des Personnes Agees (TEMPA), Wolf motor function test (WMFT), Box and Block test (BBT), and Jamar grip dynamometer, showed statistically significant progress from pretest to posttest and follow-up, indicating that the proposed system effectively promoted fine motor recovery of function. Additionally, our evidence suggests that this system was also effective under certain challenging conditions such as being in the chronic stroke phase or a coside of lesion and dominant hand (nondominant hand impaired). System usability assessment indicated that the participants strongly intended to continue using this VR-based system in rehabilitation.

## 1. Introduction

Stroke is a leading cause of long-term disability [1] with up to 76% of people with stroke experiencing a paralysis of the upper limbs at onset [2]. Although rehabilitation programs can enhance the recovery of upper-limb function, the effectiveness of therapeutic interventions is generally less pronounced in the upper limbs than in the lower limbs [3]; 55%–75% of stroke survivors continue to experience functional limitations in their upper extremities 3–6 months after a stroke [4–7]. Functional recovery in the paretic upper limbs typically exhibits a proximal-to-distal gradient, which results in hand function being poorer than arm function [8]. However, functional recovery of the upper limbs after a stroke is primarily determined by the improvement of the paretic hand. Faria-Fortini et al. [9] reported that in people with chronic stroke, grip and pinch strength are more related to upper-limb function than to proximal-end strength. Thus, efforts to improve hand and finger strength should be emphasized in rehabilitation programs. Although stroke survivors often regain most of the functional reach and grasp capabilities in their upper extremities, the recovery of the pinch skill remains incomplete in the majority of patients. Pinch movements represent an important upper-extremity motor skill [10, 11], and impaired pinch substantially affects a persons' dexterity after a stroke.

Regarding the mechanism of motor improvement, recent evidence indicates that highly repetitive and task-specific trainings may induce cortical reorganization and improve motor recovery. Functional magnetic resonance imaging- (MRI-) based research has demonstrated that VR training systems induce cortical reorganization in stroke patients [12, 13]. The mere repetition of simple tasks that lie within the capability of the performer, however, is unlikely to induce neural plasticity and learning [14]. Thus, task-oriented training programs should be (1) adequately challenging to require new learning, (2) progressive and optimally adapted to each person's capability, and (3) sufficiently stimulating to invite active participation. Creating realistic and demanding practice environments, however, is often challenging and can be limited by a facility's financial resources. Consequently, virtual reality (VR) offers numerous advantages in rehabilitation settings. VR can be used to transform tedious repetitive tasks into engaging, functional, and challenging activities, and the difficulty of tasks can be graded according to a person's ability. By providing engaging skill training and immediate appropriate and accurate performance feedback through visual and auditory rewards, virtual rehabilitation increases a persons' motivation to practice tasks [15].

Although numerous VR training systems have been developed for retraining upper-extremity functions post stroke, only a few systems have been designed to retrain hand function [16–20]. Furthermore, among the VR systems developed to retrain hand function, several designs are excessively complex for clinical use [16–18]. Installing hardware is also technically challenging, and people with stroke may require help donning the hand-tracking apparatus, which can make the process time-consuming. Moreover, the hardware used in VR systems, such as haptic devices and hand-tracking gloves, is expensive, which limits the use of these systems in rehabilitation settings. Furthermore, several VR systems do not incorporate haptics for the provision of sensory feedback [20, 21]. Sensory feedback obtained from the performance of everyday tasks is crucial for improving hand function. Incorporating haptic feedback into VR systems not only enables users to observe how they are manipulating virtual objects but also helps them experience a realistic sense of touch in the virtual environment. Moreover, VR systems integrated with haptics that provide users the feedback of proprioception can potentially help with the enhancement of motor control and muscle strength.

Novint Falcon™ (Novint Technologies Inc®, US) [22] is a commercially available, cost-effective robot arm that serves as a haptic interface. By generating forces that exhibit 3 degrees of freedom, Novint Falcon can simulate complex mechanics and produce force sensations that users perceive to be real. In this study, we employed dual-Novint Falcon devices to create a novel haptic interface to develop a stroke rehabilitation system that simulates pinch tasks and strengthens the hands and fingers to enhance the therapeutic benefit. Our objectives in this study were to (1) develop a novel haptic-enhanced VR training system for use in stroke rehabilitation, (2) determine the efficacy of the proposed system, and (3) examine the usability of the proposed system. Our hypothesis was that the proposed VR system integrated with haptics is able to improve fine motor functions effectively.

## 2. Method and Materials

### 2.1. Haptic-Enhanced Virtual Reality System

**Figure 1:**
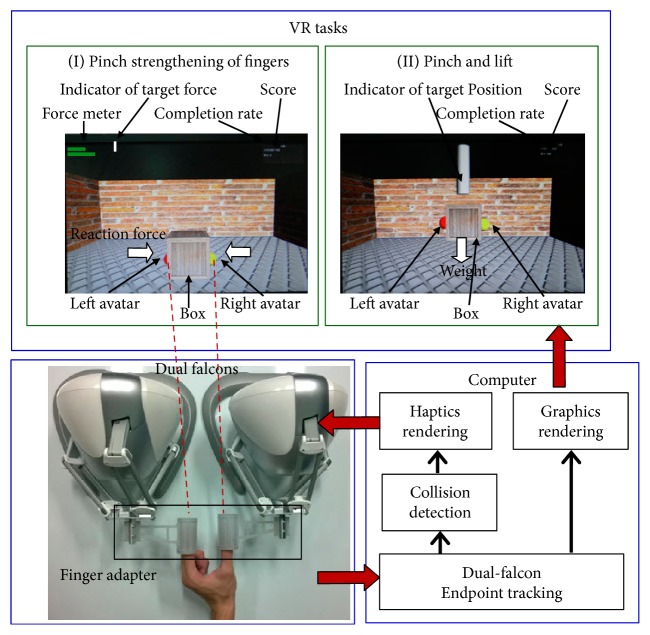
System architecture.

Developing the system described here involved implementing 3 subsystems that covered VR tasks, haptic simulation, and a user-machine interface. The system architecture is shown in Figure 1. Based on a task-oriented therapy design, the VR tasks were formulated to ensure that pinch movements were required to complete each task and that the patients experienced finger strengthening. Two VR tasks were implemented using the 3D game engine Unity™ (Unity Technologies@ US), which is a state-of-the-art toolkit used for developing 3D games deployed on multiple operating systems. The first task was a “pinch strengthening” task, in which participants were required to grip a virtual box by using 2 fingers, the thumb + each finger. The participants gradually increased their pinch strength until the default strength setting used for the simulation was achieved. The second task was a “pinch and lift” task, in which participants were required to grip a virtual box with two fingers (thumb + index and thumb + middle fingers) and lift the weighted box to a default height. In the proposed system, 2 Novint Falcon devices operated in coordination to simulate the haptic perceptions of 2 fingertips. In both tasks, the haptic simulation was implemented in such a manner that the participants perceived the reaction force of the surface and/or the weight of the box. A maximum of 20 seconds was allowed for each trial. A series of dynamically adjusted hierarchical challenges was also incorporated into the two VR tasks, based upon the severity of the participant's motor injury. In this study, we designed a user-machine interface that separately linked two of the participants' fingers with Novint Falcon, which allowed the participants to perform the pinch-skill tasks naturally, without exerting additional effort and to fully experience the effects of the haptic simulations. This user-machine interface is adaptable to various finger combinations.

### 2.2. Study Participants

We recruited 16 participants with hemiparesis and motor impairment due to stroke. Participants were included if (1) they had a first-time hemiparetic stroke recorded in the previous 2 years; (2) they obtained a physician's diagnosis of stroke that was confirmed based on the findings of neurological examinations and brain-imaging technologies (MRI or computed tomography (CT) scans); (3) they were aged between 20 and 85 years; (4) their proximal upper extremity on the more affected side was in Brunnstrom Stages II–VI; (5) they had no cognitive dysfunction, as measured using the Mini-Mental State Examination (MMSE ≧ 20) [23]; and (6) they were willing to participate in the study and sign the informed consent form. We excluded volunteers (1) with unstable vital signs; (2) who exhibited irreversible contracture of any joints of the impaired upper extremities; (3) who experienced a surgery, fracture, arthritis, or pain that may influence the recovery of upper-extremity function; (4) who exhibited spasticity (>2 on the modified Ashworth scale); (5) with uncontrolled poststroke seizures; and (6) who had experienced a heart attack in the previous 3 months. The Institutional Review Board of Taipei Veterans General Hospital, Taiwan, approved the consent procedure. The participant demographics are described in Table 1.

### 2.3. Intervention

An experienced occupational therapist supervised the haptics-based VR rehabilitation. The participants attended 30 min stroke-rehabilitation sessions three times per week for eight weeks, and all participants completed the 24 training sessions. To avoid fatigue, consecutive training sessions were conducted 24 hours apart. Each VR training session involved practicing the two VR tasks, the pinch-strengthening, and pinch-and-lift tasks. The pinch-strengthening task was performed 20 times using the thumb and each finger. The pinch-and-lift task was performed 20 times each using the thumb and the index finger, and the thumb and the middle finger. When performing the VR tasks, the participants were seated with their forearms resting on a height-adjustable table, shoulder in slight abduction, elbow at 90° flexion, and the forearm in a neutral position. Following each attempt, the occupational therapist increased the task difficulty according to the participant's abilities. As their ability increased, the level of difficulty was gradually increased. Clinical assessments were performed by an occupational therapist, with over 3 years of experience, who was not involved in treating the patients. Assessments were conducted three days before training (0 week), within three days after training (eight-week endpoint), and at a one-month follow-up session (12-week endpoint).

### 2.4. Measurements

The Fugl-Meyer assessment (FMA) [24] featuring wrist and hand portions was used to evaluate motor impairment (maximal score = 24). To measure upper-extremity function, we used the Wolf motor function test (WMFT) [25] that features four items that require distal control (*lift pencil*, *lift paper clip*, *stack checker*, and *turn a key in lock*; max = 20) and the Test Evaluant les Membres superieurs des Personnes Agees (TEMPA) [26] that features four items also involving distal control (*prehension*: *handling coins*, *picking up*, *and moving small objects*; *precision of fine motor movements*: *handling coins*, *picking up*, *and moving small objects*; −12~0). The Box and Blocks test (BBT) [27] was used to evaluate manual dexterity as measured by the number of blocks moved from one side to the next. Strength was measured using handheld dynamometers (JAMAR [28]). System usability was evaluated through Users' Technology Acceptance questionnaire [29–31], which includes dimensions of usefulness, ease of use, intention to use, and playfulness. Each item in the questionnaire was rated using a 5-point Likert scale. Finally, kinematic and kinetic data were recorded for the two fingertips using position and force in three dimensions.

### 2.5. Analysis Methods

Statistical analysis was performed on scores of clinical measurements at pretests, posttests, and follow-up in order to investigate the efficacy of the proposed system. The Wilcoxon rank sum test was applied in the statistical analysis of paired samples. The statistical tool SPSS™ was used to perform the statistical analysis.

## 3. Results

### 3.1. Clinical Measures

Efficacy is used to determine if an intervention produces the expected result under ideal (often laboratory) environments or circumstances. To investigate the efficacy of the proposed system, the scores of the pretests and posttests of the clinical measurements were analyzed (Table 2). The results indicated that the progress was statistically significant in FMA, TEMPA, WMFT, BBT, and JAMAR (all *P* < 0.05), with the progression rate of the scores being 34%, 12%, 18%, 24%, and 34%, respectively. Moreover, we analyzed the scores of the pretest and the follow-up test to assess the retention of the training obtained using the VR system presented here (Table 3); these results also indicated statistically significant progress in FMA, TEMPA, BBT, and JAMAR, with the scores progressing by 30%, 8%, 19%, and 24%, respectively. Although not significant (*P* = 0.17), the WMFT mean scores at the follow-up test were higher than those at the pretest, with the progression rate being 6%.

**Figure 2:**
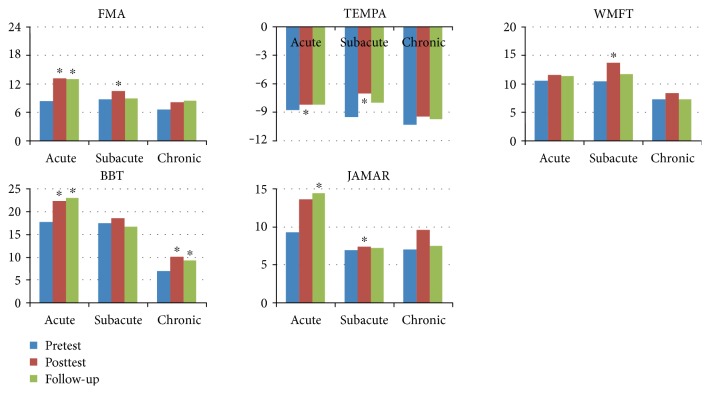
Group result: duration since stroke first hit (∗ significance of *P* value less than 0.05).

**Figure 3:**
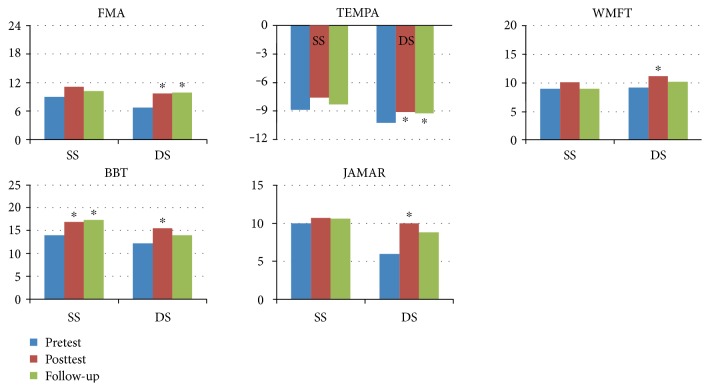
Group result: combination of lesion side and hand dominance. SS: lesion side and hand-dominance side are the same; DS: lesion side and hand-dominance side are different (∗ significance of *P* value less than 0.05).

The participants in this study were divided into groups according to their conditions so that the effect of using the VR system could be investigated in relation to each condition. First, the participants were grouped according to the duration since the first stroke occurred: acute, 0–3 months (*n* = 5); subacute, 4–6 months (*n* = 4); and chronic, 7 or more months (*n* = 7). The results of the clinical measurements obtained for each group at pretest, posttest, and follow-up are presented in Figure 2. Participants in the acute-stage group showed statistically significant progress in scores from pretest to posttest in FMA, TEMPA, and BBT, and they further showed statistically significant preservation of the training effect from pretest to follow-up in FMA, BBT, and JAMAR. Participants at the subacute stage showed statistically significant progress from pretest to posttest in the scores of FMA, TEMPA, WMFT, and JAMAR, whereas participants in the chronic stage showed statistically significant BBT progress at both posttest and follow-up with respect to pretest. Second, the participants were grouped according to the sides of lesion and hand dominance, which were either coside (right and right or left and left) or alternated side (right and left or left and right) and were denoted as same side (SS) and different side (DS), respectively (SS: *N* = 7; DS: *N* = 9). The results of the clinical measurements obtained for each group at pretest, posttest, and follow-up are presented in Figure 3. In the DS group, significant progress from pretest to posttest was observed across all 5 measures, whereas in the SS group, statistically significant progress was identified in BBT at both posttest and follow-up with respect to the pretest.

### 3.2. Synchronization of Kinematic and Kinetic Data to Interpret Behavior

**Figure 4:**
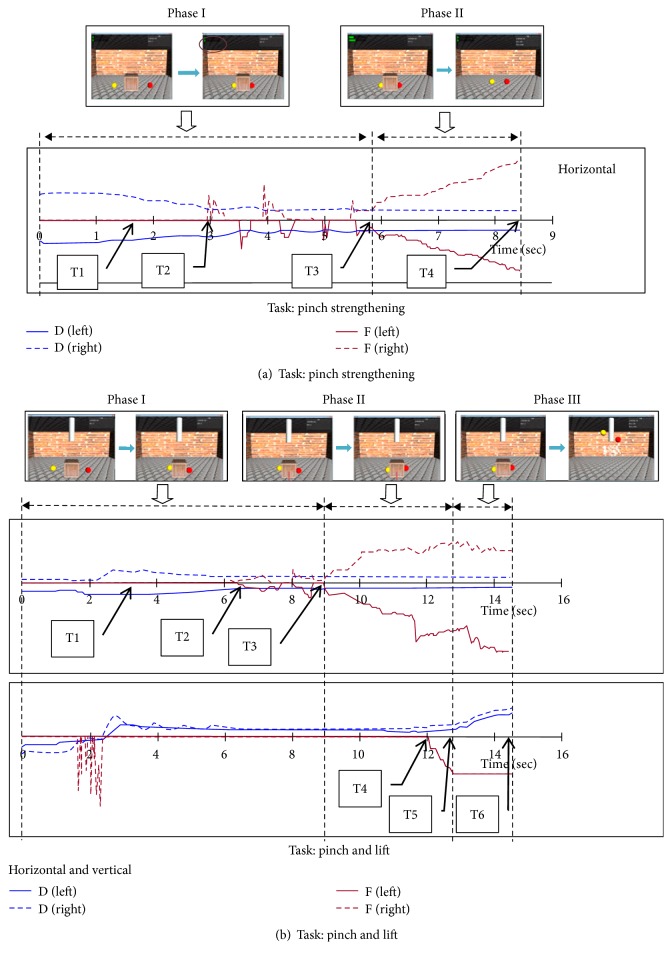
Behavior interpretation from synchronized kinematic and kinetic data being changed over time.

The kinematic data (positions in 3D) and kinetic data (force in 3D) of the two fingertips were synchronized along the same timeline to divide compound behaviors into several monobehavioral phases to facilitate their interpretation. A sample dataset acquired while participants were performing the two VR tasks is presented in Figure 4. In the pinch-strengthening task, two monobehavioral phases were detected (Figure 4(a)). The first phase started at the beginning of the task and ended when the two fingers were concurrently in contact with the target cube and the reaction forces were perceived to be horizontal. At this point, the second phase began, in which the participant gradually increased the strength of the two fingers until the strength required for this trial was reached. In the pinch-and-lift task, three monobehavioral phases were detected (Figure 4(b)). The first phase was similar to the first phase in the pinch-strengthening task. During the second phase, the participant gradually increased the pinch strength to enhance the friction applied and overcome the cube weight so as to lift the target cube, marking the end of the second phase. While experiencing the reaction force and the cube weight during the third phase, the participant gradually moved the target cube upward until it reached the required height set in this trial.

### 3.3. System Usability

The results of system usability evaluation, which featured four dimensions, were all higher than 4.0 points (Table 4), indicating that the participants considered the system to be useful, playful, easy to use, and that they strongly intended to continue using this VR system in rehabilitation.

## 4. Discussion

The key element addressed in this system was the haptic-enhanced simulation that was integrated with VR tasks to facilitate fine motor rehabilitation. We focused on 3 questions when conducting this study: (1) what are the types of haptic system proposed previously to support fine motor rehabilitation? This addressed whether the type of system developed here was novel; (2) how might the haptic system be applied to the upper extremity while working coordinately with VR? This addressed how the haptics applied in this study might be more effective in fine motor rehabilitation than that which has been applied previously; and (3) what is the cost of existing haptic systems that support fine motor rehabilitation? This addressed whether the system developed in this study has the potential to be used widely in clinics.

Regarding the types of haptic system used for fine motor rehabilitation in previous studies, diverse haptic systems were proposed, in which force sensation was provided using a pneumatic glove [32, 33], a hand exoskeleton [16, 34, 35], a full-scale arm-like robotic exoskeleton [35–37], a cable-enabled glove [33], or a hand-held stylus [38]. Compared with the designs used in these studies, the design of our system and the included VR tasks is novel and distinct because the design integrated *dual* robot arms that required 2 fingers to work synchronously (i.e., the dual robot arms worked coordinately), and therefore the pinch task had to be completed precisely. Regarding the manner in which haptic systems are applied to the upper extremity while working with VR, 3 conditions can be identified in previous studies: (1) no haptics (pure VR) [39]; (2) haptics applied to the arm [35–37]; and (3) haptics applied to the fingers or the hand [32–35, 38]. In the case where no haptics was applied [39], statistically significant progress was reported in the FMA scores of stroke patients specifically in the acute phase. However, our results showed significant progress in FMA scores of patients in all 3 phases: acute (*N* = 5), subacute (*N* = 4), and chronic (*N* = 7); thus, the proposed system featuring force sensation appears to be more effective in treating stroke populations of all phases than previous systems were in doing so. In the cases in which haptics was applied to the arm, in one study [36], statistically significant progress in WMFT was reported, but the study lacked the other measurements obtained in our study. Another study [37] reported no significant progress in WMFT and BBT, but we measured significant progress in these scores in our study. Based on these results, we conclude that a system in which haptics is applied to fingers might be more effective when used for fine motor rehabilitation, as compared with a system in which haptics is applied to the arm only. Lastly, with regard to haptics being applied to the hand or fingers, previous studies conducted using a hand exoskeleton [16, 34, 35] and a hand-held exoskeleton [38] reported results in the form of descriptive statistics. However, one study [32] reported significant progress in FMA and BBT scores, which agrees with the results of our study. Compared with the aforementioned studies, the results of this study provide stronger evidence (statistically significant progress in FMA, TEMPA, WMFT, BBT, and JARMAR) supporting the view that haptics applied to fingers, as in the case of the system presented here, might be more effective when used in fine motor rehabilitation than haptics applied to the arm alone. Regarding the cost issue, the haptic system presented here costs less than US$ 500, which is considerably less than the cost of systems used in the studies listed in the preceding paragraph. Furthermore, the robot arm Novint Falcon is an off-the-shelf product that can be purchased from numerous retailers. Thus, our new VR system can potentially be used more widely than other systems in clinics and even in home-based applications.

In addition to the overall results of the participants discussed thus far, we collected results after dividing the participants into groups based on a variety of selected conditions to compare the effects of using the system within groups or between groups under specific conditions. The first condition selected was the duration since the first hit of stroke. From a clinical perspective, progress made in the acute phase of stroke is considered to be more apparent than that in other phases because of spontaneous recovery. This agrees with our results because we identified statistically significant progress from pretest to posttest and follow-up across all measures except WMFT. By contrast, the progress in the chronic phase is generally considered to be less obvious than that in other phases. However, the BBT results obtained in this study showed that statistically significant progress occurred in the chronic phase from the pretest to both the posttest and the follow-up. Among the 5 clinical measures, BBT is considered to be the most relevant to hand functions, and thus our results suggest that the proposed system facilitates the recovery of fine motor function in stroke patients in the chronic phase. The second condition was the combinations of lesion side and hand-dominance side. Clinically, progress is typically considered to be more definitive if the lesion side is distinct from the side of hand dominance than if the sides are the same, because the paretic side is the same as the hand-dominance side that exhibits superior functions before the stroke. This agrees with our results because statistically significant progress from pretest to posttest was identified across all measures. Conversely, progress may not be readily discernible if the lesion side is the same as the side of hand dominance. However, our results showed statistically significant progress in BBT scores from pretest to both posttest and follow-up when the lesion side was the same as the hand-dominance side. This result indicates that the proposed system may contribute to the progress of fine motor function in patients with lesion and hand dominance on the same side.

Based on our results, we also conclude that using the proposed system featuring haptic simulations applied directly to fingers and repetitively practicing the pinch tasks may be effective in treating a patient with harsh conditions such as a chronic stroke phase or a coside of lesion and hand dominance. The 16 stroke patients who were recruited in this clinical trial completed 24 30 min training sessions without complaints, and the system presented no severe technical setbacks, indicating that the rehabilitation system can be used effectively and safely. The participants' acceptance scores of the proposed system were high, and interviews conducted with all participants confirmed their acceptance of this new technology applied in stroke rehabilitation. One of the participants was apprehensive nervous when performing the VR tasks for the first time, but showed an increase in confidence upon noticing progress. Another participant reported becoming completely engaged in the tasks and noticing pain less during the tasks, because a substantial amount of attention was devoted to enjoying playing the VR games.

A limitation of the study is that the number of participants (16) recruited in the clinical trial was small; consequently, the preliminary results presented in this paper must be further verified by performing clinical trials of a comparatively larger scale. Moreover, the design of this study hinged on the capabilities of the 2 robot arms, such as the maximal force output, the working zone, and the degrees of freedom, which limited the diversity of the tasks that could be developed.

## 5. Conclusion and Future Research

In this paper, we have described a novel haptic-enhanced VR system featuring haptic simulation that was developed to facilitate the long-term poststroke recovery of upper-extremity motor function. The results of the clinical trial performed using this system showed that all clinical measures examined (FMA, TEMPA, FMA, BBT, and JAMAR) exhibited statistically significant progress, indicating that the proposed system effectively promotes fine motor rehabilitation. Specifically, the results presented here reveal that this new VR system is also effective in treating certain harsh conditions such as a chronic stroke phase and a coside of lesion and hand dominance. By synchronizing kinematic and kinetic data, various behavioral phases are clearly interpreted. Moreover, measures of system usability indicate that the participants in this study strongly intend to continue using the proposed system in rehabilitation. In future work, a clinical trial larger than the one described here could be used to verify the preliminary results of this study. Furthermore, the prosperous rich kinematic and kinetic data gathered in clinical trials could be analyzed using advanced computational techniques to develop new assessment methods.

## Figures and Tables

**Table 1:** Demographic data of each participant.

ID	Sex	Age	Stroke duration (month)	Hand dominance	Type of stroke	Lesion side	Lesion location	Brunnstrom stage (distal)
1	F	82	1	R	Ischemic	L	MCA	3
2	M	72	15	R	Ischemic	R	PCA	3
3	M	68	12	R	Hemorrhage	R	PCA	4
4	M	53	2	R	Hemorrhage	L	MCA	2
5	M	59	1	R	Ischemic	L	MCA	5
7	M	39	16	R	Hemorrhage	R	MCA	3
8	M	60	18	R	Hemorrhage	R	MCA	2
9	M	65	8	R	Ischemic	R	MCA	4
10	F	38	5	R	Hemorrhage	R	PCA	4
11	M	58	4	R	Ischemic	L	MCA	5
13	F	44	11	L	Ischemic	R	MCA	4
15	F	61	4	R	Ischemic	L	MCA	4
16	F	33	6	R	Ischemic	L	MCA	2
33	M	28	14	L	Hemorrhage	R	MCA	3
34	M	69	3	R	Hemorrhage	R	PCA	6
35	F	60	1	R	Ischemic	L	MCA	2

**Table 2:** Results of Wilcoxon rank sum test on clinical measures: pretest versus posttest (*N* = 16).

Measure	Pretest mean (SD)	Posttest mean (SD)	*z* value	Significance
FMA	7.69 (5.97)	10.31 (7.35)	−2.994	<0.01
TEMPA	−9.63 (2.75)	−8.44 (3.29)	−2.422	<0.01
WMFT	9.13 (5.55)	10.75 (4.77)	−2.283	<0.05
BBT	13.00 (13.68)	16.06 (15.06)	−2.810	<0.01
JAMAR	7.71 (6.48)	10.36 (7.81)	−1.707	<0.05

**Table 3:** Results of Wilcoxon rank sum test on clinical measures: pretest versus follow-up (*N* = 16).

Measure	Pretest mean (SD)	Follow-up mean (SD)	*z* value	Significance
FMA	7.69 (5.98)	10.00 (7.29)	−2.455	<0.01
TEMPA	−9.63 (2.75)	−8.81 (3.47)	−1.916	<0.05
WMFT	9.13 (5.55)	9.68 (4.6)	−0.954	0.17
BBT	13.00 (13.68)	15.44 (13.93)	−2.242	<0.05
JAMAR	7.71 (6.48)	9.59 (7.42)	−1.733	<0.05

**Table 4:** Results of user usability evaluation.

	Usefulness	Playfulness	Intention to use	Ease of use
Mean (SE)	4.35 (0.55)	4.47 (0.61)	4.85 (0.28)	4.19 (0.53)
